# Effect of temperature on *Escherichia coli* bloodstream infection in a nationwide population-based study of incidence and resistance

**DOI:** 10.1186/s13756-022-01184-x

**Published:** 2022-11-23

**Authors:** Sarah F. Feldman, Elizabeth Temkin, Liat Wulffhart, Amir Nutman, Vered Schechner, Pnina Shitrit, Racheli Shvartz, Mitchell J. Schwaber, Yehuda Carmeli

**Affiliations:** 1 National Institute for Antibiotic Resistance and Infection Control, Ministry of Health, Tel Aviv, Israel; 2 Sackler Faculty of Medicine, Tel Aviv University, Tel Aviv, Israel; 3 Infection Control Unit, Meir Medical Center, Kefar Sava, Israel

**Keywords:** *Escherichia coli*, Bloodstream infection, Antibiotic resistance, Epidemiology, Temperature, Seasonal variation

## Abstract

**Background:**

The incidence of *Escherichia coli* bloodstream infections (BSI) is high and increasing. We aimed to describe the effect of season and temperature on the incidence of *E. coli* BSI and antibiotic-resistant *E. coli* BSI and to determine differences by place of BSI onset.

**Methods:**

All *E. coli* BSI in adult Israeli residents between January 1, 2018 and December 19, 2019 were included. We used the national database of mandatory BSI reports and outdoor temperature data. Monthly incidence and resistance were studied using multivariable negative binomial regressions with season (July–October vs. other) and temperature as covariates.

**Results:**

We included 10,583 events, 9012 (85%) community onset (CO) and 1571 (15%) hospital onset (HO). For CO events, for each average monthly temperature increase of 5.5 °C, the monthly number of events increased by 6.2% (95% CI 1.6–11.1%, *p* = 0.008) and the monthly number of multidrug-resistant events increased by 4.9% (95% CI 0.3–9.7%, *p* = 0.04). The effect of season was not significant. For HO events, incidence of BSI and resistant BSI were not associated with temperature or season.

**Conclusion:**

Temperature increases the incidence of CO *E. coli* BSI and CO antibiotic-resistant *E. coli* BSI. Global warming threatens to increase the incidence of *E. coli* BSI.

**Supplementary Information:**

The online version contains supplementary material available at 10.1186/s13756-022-01184-x.

## Introduction

The impact of *Escherichia coli* bloodstream infection (BSI) is high in terms of incidence, antibiotic resistance, and mortality [1, 2]. A global increase in the incidence of *E. coli* BSI has been described [3, 4], with seasonal variations: the incidence rises with the warm season [5–10] and with increased temperature [4, 6, 8, 10]. Explanations for the seasonality of the incidence of *E. coli* BSI include higher *E. coli* colonization in humans during the summer because of behavior changes and modification of host immunity [10, 11], and increased virulence and growth of *E. coli* in food and in the environment [6, 10–12].

Several aspects of seasonality in *E. coli* BSI require further assessment. It is unclear whether seasonality in the incidence of *E. coli* BSI differs between community-onset (CO) events and hospital-onset (HO) events [7–9]. It is unknown if seasonality affects resistance. To our knowledge, only one study was conducted, in Oxfordshire, England in 1999–2011, and found a non-significant increase in the incidence of resistant events at higher temperatures [4].

The aim of the present study was to describe the effect of season and outdoor temperature on the incidence of *E. coli* BSI and antibiotic-resistant *E.* *coli* BSI, by place of onset.

## Methods

### Data sources

All acute care hospitals in Israel send mandatory monthly reports of BSI caused by sentinel pathogens, including *E. coli*, to the National Institute for Antibiotic Resistance and Infection Control, as described previously [2]. Those reports include the patient’s name, date of birth, national identity number, age, sex, admission date, ward, sample date, pathogens isolated, and results of antibiotic susceptibility testing. Data were de-identified for the purposes of this study. We used publicly available outdoor temperature data from the Ministry of Transportation [13]. We calculated the monthly mean of daily maximum outdoor temperature using data from January 1, 2018, to December 31, 2019 from four stations in Israel (Haifa, Tel Aviv, Jerusalem, and Mitzpe Ramon).

### Study sample

All *E. coli* BSI that occurred between January 1, 2018 and December 31, 2019 in patients aged 18 and over were included in the analysis. We excluded events with a missing or implausible admission date and thus an undefined place of onset.

### Definitions

An *E. coli* BSI event was defined as a blood culture positive for *E. coli*. The date of onset of the event was the date of blood sample collection. If a blood culture was positive for another sentinel pathogen (*Acinetobacter baumannii*, *Enterococcus faecalis*, *Enterococcus faecium*, *Klebsiella pneumoniae*, *Pseudomonas aeruginosa*, *Staphylococcus aureus*, or *Streptococcus pneumoniae*) within 5 days before or after an *E. coli* BSI event, the event was defined as polymicrobial. An event was defined as CO if it occurred within the first 3 days of hospital admission, and as HO otherwise. We defined seasons as winter (December–February), spring (March–May), summer (June–August), and autumn (September–November). We also modelled season dichotomously as holiday season (July–October), which corresponds in Israel to both a hot period and a period when many people are on vacation, vs. the rest of the year. *E. coli* was classified according to the hospitals’ laboratory reports as susceptible, intermediate or resistant to a given antibiotic. We grouped together isolates that were intermediate or resistant. Multidrug resistance (MDR) was defined as resistance to three or more antimicrobial classes [14].

### Statistical analysis

No sample size calculations were performed for this population-based study; we included all cases of BSI in adults during the study period. Variables were described by mean and standard deviation (SD) or by count and percentage, as appropriate. The mean number of events per month was compared between seasons using negative binomial regression. Age was compared between seasons using Student’s t-test or ANOVA. Categorical variables were compared between seasons using the chi-square test of independence.

Seasonal variation was assessed by fitting a sinusoidal curve to the total monthly number of events and computing the peak-to-low ratio (i.e., the ratio of the peak monthly number of events to the lowest monthly number of events on the sinusoidal curve) and its 95% confidence interval (CI). The peak-to-low ratio was calculated using moment-based estimation, with non-transformed data and a second-order moment statistic, as previously described [15].

The monthly number of events was standardized and plotted by temperature. The monthly number of CO events was plotted by temperature and by age category. A simple linear model of the monthly number of CO events by temperature was computed in each age category and the coefficient of temperature was plotted. The monthly number of events by temperature was plotted by MDR status and was fitted using a third degree polynomial. The monthly number of events and the monthly number of resistant events were modeled using negative binomial regression. CO and HO events were studied separately in all models. The explanatory variables were season (holiday season vs. rest of the year), temperature, sex, age category and the number of months since January 2018 to account for an increasing trend in incidence. Incidence rate ratio (IRR), 95% CI and* p* values were calculated. Variables were selected based on knowledge and availability, without a statistical selection process.

The monthly peak-to-low ratio, its 95% CI and the time of peak were computed using Episheet (https://www.drugepi.org/dope/software#Episheet). All other analysis were performed using R version 4.0.4.

## Results

### Effect of season on incidence

We analyzed 10,583 *E. coli* BSI events in 9733 patients, after exclusion of 530 events with an unknown place of onset. A total of 9012 (85.2%) events were CO and 1571 (14.8%) were HO. The mean age was lower during the holiday season for CO events (72.6 [SD 17.0] in the holiday season vs. 73.9 [SD 16.4] during the rest of the year; *p* < 0.001) but was similar in the two periods for HO events (*p* = 0.6) (Table 1).

**Table 1:** Characteristics of *E. coli* bloodstream infections by place of onset and by season

Variable	Community onset			Hospital onset		
	(n = 9012)			(n = 1571)		
	Holiday season	Rest of the year	*P*	Holiday season	Rest of the year	*P*
*E. coli* BSI per month, mean (SD)	410.5 (29.6)	357.9 (35.3)	< 0.001	69 (8.8)	63.7 (7.7)	0.13
Age, mean (SD)	72.6 (17.0)	73.9 (16.4)	< 0.001	67.1 (17.0)	67.6 (17.1)	0.56
Age category, N (%)			< 0.001			0.73
18–44	268 (8.2)	396 (6.9)		66 (12.0)	120 (11.8)	
45–64	527 (16.0)	870 (15.2)		130 (23.6)	230 (22.6)	
65–74	736 (22.4)	1158 (20.2)		154 (27.9)	267 (26.2)	
75 +	1753 (53.4)	3304 (57.7)		202 (36.6)	402 (39.5)	
Female sex, N (%)	1855 (56.5)	3362 (58.7)	0.04	270 (48.9)	507 (49.8)	0.75
Polymicrobial event, N (%)	200 (6.1)	370 (6.5)	0.49	79 (14.3)	153 (15.0)	0.71

Holiday season: July to October

*SD* Standard deviation

**Fig. 1:**
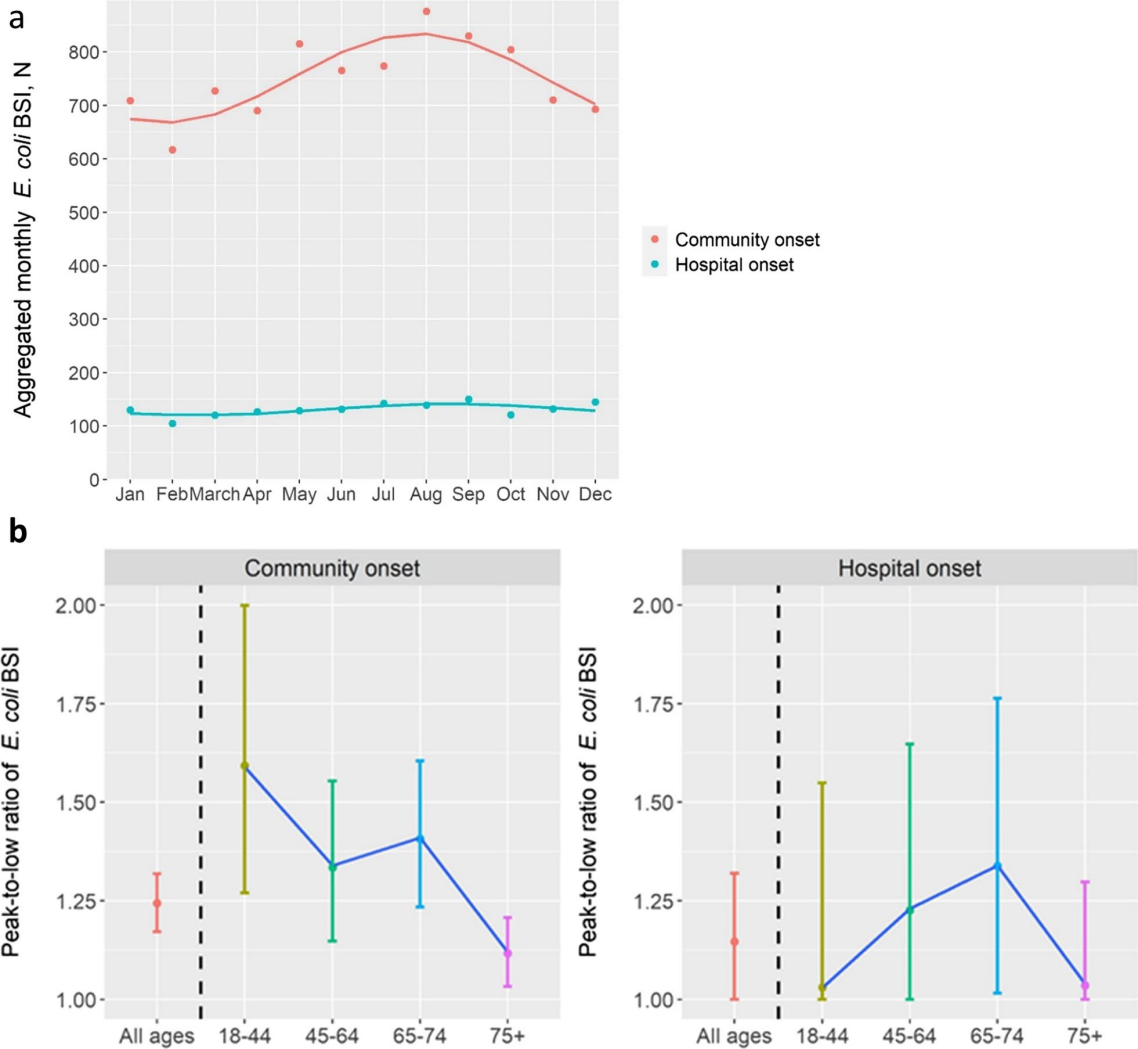
Seasonality of *E. coli* bloodstream infections (BSI), by place of onset. **a** Each dot represents the total observed number of *E. coli* BSI each month. The lines represent the fitted number of *E. coli* BSI, using a periodic model. **b** Peak-to-low ratio (ratio of the peak monthly number of events to the lowest monthly number of events on the sinusoidal curve) and its 95% confidence interval in all ages and by age category

Figure 1 A shows the number of *E. coli* BSI per month fitted with a periodic model. Seasonality was observed for CO events (Fig. 1 A) and the peak-to-low ratio was 1.24 (95% CI 1.17–1.32) for all ages combined (Fig. 1B) with the peak date on August 10. For HO BSI, no seasonality was observed (Fig. 1 A) with a peak-to-low ratio of 1.15 for all ages combined (95% CI 1.00–1.32) (Fig. 1B) and the peak date on September 2. The seasonality of CO events was stronger in younger age categories than in the 75 + category (Fig. 1B). For CO BSI, the monthly mean number of events was higher in the holiday season than in the rest of the year (410.5 [SD 29.6] vs. 357.9 [SD 35.3], *p* < 0.001) (Table 1). When season was modeled as 4 categories, the monthly mean number of CO events increased from 336.5 in winter (SD 27.7) to 372.0 (SD 38.9) in spring, 402.5 (SD 35.4) in summer, and then declined slightly in autumn to 390.7 events (SD 37.4) (*p* = 0.008) (Additional file 1: Table S1).

### Effect of temperature on incidence

**Fig. 2:**
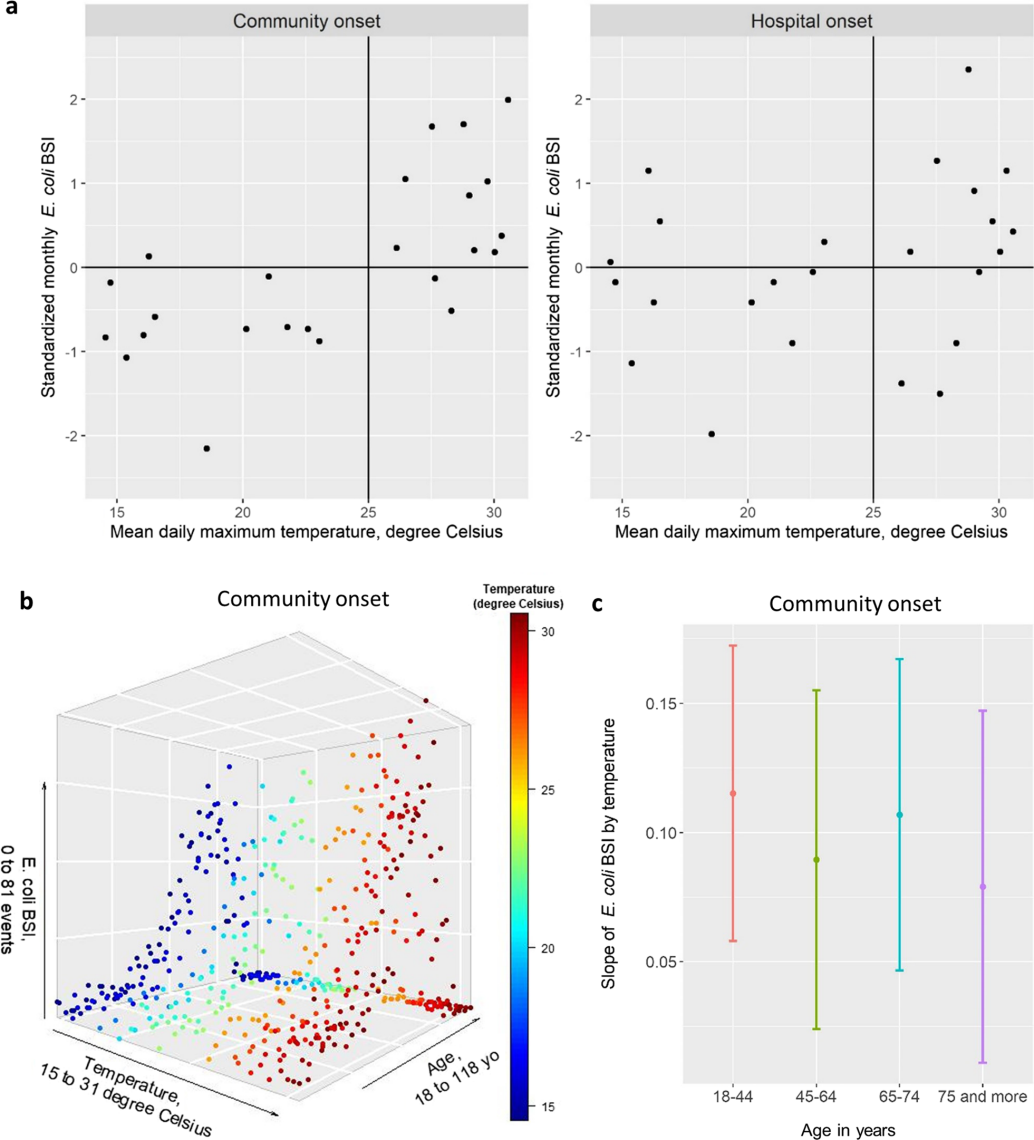
Association between the incidence of *E. coli* bloodstream infection (BSI) and temperature. **a** Each dot represents a month. **b** Each dot represents a month in a specific 5-year age category. **c** Coefficient and 95% confidence interval of the simple linear model of the number of events by temperature

Figure 2 A shows that, among CO *E. coli* BSI, the monthly number of events increased with temperature only above 25 °C; no correlation was observed among HO BSI. The effect of temperature on the monthly number of CO BSI was stronger in younger age groups (Fig. 2B, C). Table 2 shows the multivariable models of the incidence of *E. coli* BSI events by place of onset. Among CO events, the monthly number of events increased significantly by 1% per 1 °C, or 6.2% (95% CI 1.6–11.1%) for each 5.5 °C increase (*p* = 0.008); but season (modelled dichotomously) was not significantly associated with BSI incidence. Among HO events, no significant association was found with temperature nor season.

**Table 2:** The effect of season and temperature on the incidence of *E. coli* bloodstream infection, by place of onset

Variable	Community onset			Hospital onset		
	IRR	95% CI	*P*	IRR	95% CI	*P*
Holiday season	1.07	0.97, 1.18	0.2	1.03	0.88, 1.19	0.7
Temperature, °C*	1.01	1.00, 1.02	0.008	1.00	0.99, 1.02	0.6
Female sex	1.42	1.33, 1.52	< 0.001	0.98	0.88, 1.08	0.7
Age category, years						
18–44	–	–		–	–	
45–64	2.18	1.94, 2.44	< 0.001	1.93	1.62, 2.32	< 0.001
65–74	2.97	2.65, 3.32	< 0.001	2.26	1.90, 2.70	< 0.001
75 +	7.84	7.05, 8.72	< 0.001	3.25	2.75, 3.85	< 0.001
Number of months since January 2018	1.01	1.00, 1.01	0.02	1.01	1.00, 1.02	0.01

*When temperature was modelled dichotomously as ≤ 25 °Cor > 25 °C, results were IRR 1.16 (95% CI 1.05, 1.27) *p* = 0.002 for CO BSI and 1.03 (95% CI 0.89, 1.19) *p* = 0.7 for HO BSI

Holiday season: July to October

*IRR* Incidence rate ratio, *CI* confidence interval

### Antimicrobial resistance

**Fig. 3:**
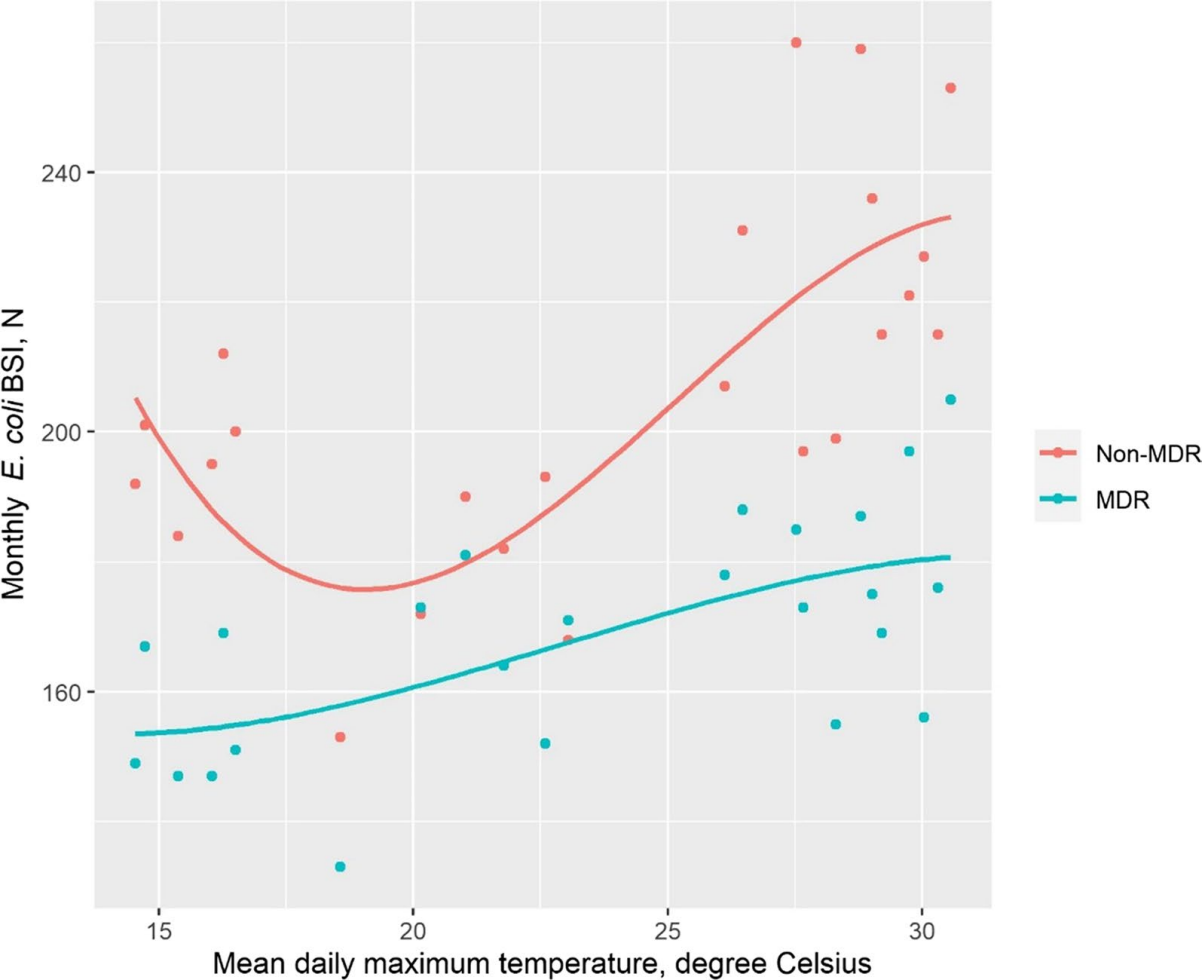
Community-onset *E. coli* bloodstream infections by multidrug-resistance (MDR) status and by temperature

As shown in Table 3, among CO BSI the number of MDR events significantly increased by 1% per 1 °C, or 4.9% (95% CI 0.3–9.7%) for each  5.5 °C increase (*p* = 0.04). Season (modelled dichotomously) was not significantly associated with the incidence of MDR BSI. Among HO events, there was no significant association between the incidence of MDR BSI and temperature or season. Non-MDR CO events increased only at temperatures above 25 °C, while MDR CO events started to increase at temperatures above 20 °C (Fig. 3).

**Table 3:** Multivariable analysis of the effect of season and temperature on the incidence of multidrug-resistant *E. coli* bloodstream infection, by place of onset

Variable	Community onset			Hospital onset		
	IRR	95% CI	*P*	IRR	95% CI	*P*
Holiday season	1.04	0.94, 1.14	0.50	1.00	0.84, 1.20	> 0.9
Temperature, °C	1.01	1.00, 1.02	0.04	1.01	0.99, 1.02	0.30
Female sex	1.10	1.03, 1.18	0.01	0.84	0.74, 0.95	0.01
Age category, years						
18–44	–	–		–	–	
45–64	3.03	2.58, 3.56	< 0.001	1.75	1.41, 2.18	< 0.001
65–74	4.16	3.57, 4.88	< 0.001	2.04	1.65, 2.53	< 0.001
75 +	11.4	9.90, 13.3	< 0.001	2.62	2.14, 3.23	< 0.001
Number of months since January 2018	1.01	1.00, 1.01	0.03	1.01	1.00, 1.02	0.14

Holiday season: July to October

*IRR* Incidence rate ratio, *CI* confidence interval

Table 4 summarizes the effects of season and temperature on the incidence of BSI caused by *E. coli* resistant to specific antibiotics. Among CO events, as temperatures rose, there was a significant increase in the incidence of BSI caused by *E. coli* resistant to amoxicillin-clavulanic acid or ampicillin-sulbactam, third- or fourth-generation cephalosporins, fluoroquinolones, and MDR *E. coli* (IRR: 1.01, 95% CI 1.00–1.02 for all); the effect of season was not significant. Among HO events, no significant association was found between the incidence of resistant BSI and temperature or season.

**Table 4:** Multivariable analysis of the effect of season and temperature on the incidence of bloodstream infection caused by *E. coli* with specific antibiotic-resistant profiles, by place of onset

Resistance phenotype	Variable	Community onset				Hospital onset			
		Resistant/total tested	IRR	95% CI	*P*	Resistant/total tested	IRR	95% CI	*P*
Amoxicillin-clavulanic acid or ampicillin-sulbactam		1020/3009				445/963			
	Holiday season		1.00	0.90, 1.11	> 0.9		1.07	0.87, 1.33	0.5
	Temperature, °C		1.01	1.00, 1.02	0.03		1.01	0.99, 1.03	0.4
3rd- or 4th-generation cephalosporins		964/3236				456/1003			
	Holiday season		0.94	0.85, 1.05	0.30		0.96	0.78, 1.20	0.7
	Temperature, °C		1.01	1.00, 1.02	0.01		1.00	0.98, 1.02	0.8
Fluoroquinolones		1116/3266				495/1014			
	Holiday season		0.97	0.88, 1.08	0.60		1.04	0.84, 1.28	0.7
	Temperature, °C		1.01	1.00, 1.02	0.02		1.00	0.98, 1.02	0.8

All models were adjusted for sex, age category and number of months since January 2018

Holiday season: July to October

*IRR* Incidence rate ratio, *CI* confidence interval

## Discussion

Our nationwide study showed a seasonal and temperature effect on the incidence of CO *E. coli* BSI and CO antibiotic-resistant *E. coli* BSI, but not on HO *E. coli* BSI. The incidence of CO *E. coli* BSI increased by 6.2% for each 5.5 °C increase. Similar results were previously found [4, 6], however, without distinction by place of onset. We found a peak-to-low ratio of 1.24 for CO events, equal to the ratio previously found for community-acquired *E. coli* BSI events in a Danish study conducted in 2000–2011 [5]. Various explanations have been suggested for this association. Season affects human behavior, with summertime increases in outdoor activity, travel, sexual activity, and changes in food consumption that increase both exposure to the environment and inter-human exposure [11, 16, 17]. Higher temperature increases the growth of *E. coli* in vitro [12], its density in the environment [6, 11], and contamination of food and beverages, leading to an increase in *E. coli* BSI, possibly through an increase in the incidence of colonization [11]. Temperature could also increase the risk of infection by possibly affecting virulence factors of *E. coli* and host immunity [10, 11].

In our study, the effect of temperature and season varied with age category; it was strongest in 18–44 year olds and smaller in the oldest age group. In contrast, Gradel et al. found that the effect of seasonality on *E. coli* BSI did not vary by age and also found little difference in the seasonal variation of the primary source of infection (e.g., urinary tract, intra-abdominal) [5]. Gradel’s study was limited by missing data for the primary source of infection in many cases. The differential effect of seasonality by age found in our study may be explained by a difference in the cause of *E. coli* BSI. Among 18–44 year olds, *E. coli* BSI is likely to be secondary to urinary tract infection through sexual activity or intra-abdominal infection through travel and outdoor activities, both varying with season [16, 17], while in the oldest age group *E. coli* BSI may develop from urinary tract infections caused by urinary devices and urinary retention, the use of invasive devices, and institutionalization [18].

We did not find seasonality in HO *E. coli* BSI, in accordance with previous studies [7, 9]. One study conducted in Belgium 2000–2014, on several pathogens including *E. coli*, did report an association between temperature and hospital-acquired BSI [8]. The results of that study might differ from ours because the seasonality of BSI differs between pathogens [5] and because in-hospital climate control may differ between countries. The absence of correlation between outdoor temperature and HO *E. coli* BSI in the climate-controlled Israeli hospitals suggests that temperature control may be an important infection prevention measure in hospitals.

We found that the monthly number of CO MDR *E. coli* BSI was associated with temperature, with a 4.9% increase in MDR *E. coli* BSI for each 5.5 °C rise in outdoor temperature. A study conducted in Oxfordshire, England, between 1999 and 2011 of 2240 *E. coli* BSI reported a non-significant positive trend between the incidence of resistant events and temperature, regardless of place of onset [4]. A large study of 28 European countries from 2000 to 2016 examined the link between outdoor temperature and the percentage of resistance among several pathogens, including *E. coli*, in various types of infections [19]. The authors found that a higher annual average minimum outdoor temperature was associated with a higher percentage of *E. coli* infections that were antibiotic resistant, and a higher rate of increase in the percentage of resistance. Several mechanisms could explain the association between resistance and temperature: carriage of extended-spectrum ß-lactamase-producing *E. coli* is associated with higher temperature [20], and horizontal gene transfer is temperature dependent [21, 22], with higher temperature enhancing cell-to-cell plasmid transfer in *E. coli* [23]. In the next decades, global warming could further increase the incidence of MDR *E. coli* BSI.

The strengths of our study were its nationwide scale and the fact that we considered place of BSI onset when studying the effects of seasonality and temperature. Our study had several limitations. First, our data were limited to two years, which diminished the power of our analysis and prevented us from defining more precisely a cut-off for the effect of temperature. Second, CO BSI included both healthcare-associated and true community-acquired events; because the anatomic source of BSI differs between those two types of acquisition, it would be interesting to study seasonality of those events separately. Third, because we had no data on behavioral factors or source of infection, we could not test whether behavioral factors could explain the seasonality. Fourth, Israel has a small range of temperatures and little difference between seasons; our study should be replicated in a country with greater weather variability to see if our findings are confirmed.

## Conclusion

We found that a higher temperature was associated with an increase in total and resistant CO *E. coli* BSI. Global warming could further increase the incidence of *E. coli* BSI.

## Supplementary Information

**Additional file 1: Table S1**. Characteristics of *E. coli* bloodstream infections by place of onset and by season.

## Data Availability

The National Institute for Antibiotic Resistance and Infection Control’s bloodstream infection database is a governmental database to which access is restricted by Israeli regulations. Computer code in R and Episheet is available from the authors upon request.

## Abbreviations

BSI: Bloodstream infection
HO: Hospital-onset
CO: Community-onset
MDR: Multidrug resistance
