# Supplementary material for: Effect of temperature on Escherichia coli bloodstream infection in a nationwide population-based study of incidence and resistance
Source: Antimicrob Resist Infect Control. 2022 Nov 23;11:144. doi: 10.1186/s13756-022-01184-x (PMC9685946; doi:10.1186/s13756-022-01184-x)
Supplement: Supplementary file 1 — Additional file 1: Table S1. Characteristics of E. coli bloodstream infections by place of onset and by season. [file 13756_2022_1184_MOESM1_ESM.pdf]

Table S1. Characteristics of *E. coli* bloodstream infections by place of onset and by season

	Community onset					Hospital onset				
Variable	Winter	Spring	Summer	Autumn	P	Winter	Spring	Summer	Autumn	P
E. coli BSI per month, mean (SD)	336.5 (27.7)	372.0 (38.9)	402.5 (35.4)	390.7 (37.4)	0.008	63.3 (9.5)	62.7 (7.7)	68.7 (6)	67.2 (10.1)	0.51
Age, mean (SD)	74.5 (15.8)	73.7 (16.6)	72.6 (17)	73.3 (16.8)	0.002	68.2 (16.9)	67.7 (17)	67.7 (16.7)	66.3 (17.7)	0.44
Female sex, N (%)	1218 (60.3)	1281 (57.4)	1378 (57.1)	1340 (57.2)	0.10	197 (51.8)	169 (44.9)	205 (49.8)	206 (51.1)	0.22
Polymicrobial event, N (%)	124 (6.1)	136 (6.1)	140 (5.8)	170 (7.3)	0.18	46 (12.1)	66 (17.6)	58 (14.1)	62 (15.4)	0.19

SD: standard deviation
